# Immunophenotype

**DOI:** 10.1186/1546-0096-12-S1-I10

**Published:** 2014-09-17

**Authors:** Salvatore Albani

**Affiliations:** 1 SingHealth Translational Immunology and Inflammation Centre, SingHealth - Duke-NUS Graduate Medical School, Singapore, Singapore

## 

The evolution of cellular and molecular immunology has made available high-throughput discovery tools which can provide an entirely new, comprehensive and multi-dimensional picture of the immune system. A combination of different approaches, such as deep phenotyping by mass and flow cytometry, multiplex gene expression and functional assays, have been applied to identify immunological and epigenetic signatures leading to the prediction of responsiveness to anti-TNF therapy in autoimmune arthritis. These signatures are originated at the microenvironmental interface between the immune system and the target organ and can then be found in the periphery. We will present novel data demonstrating this concept in the context of human autoimmune arthritis.

## Disclosure of interest

None declared.

